# Well leg compartment syndrome following robot-assisted radical cystectomy in the lithotomy position: a case report

**DOI:** 10.1186/s40981-021-00414-2

**Published:** 2021-01-28

**Authors:** Masataka Fukuda, Izumi Kawagoe, Tsukasa Kochiyama, Nozomi Ando, Osamu Kudoh, Daizoh Satoh, Masakazu Hayashida

**Affiliations:** Department of Anesthesiology and Pain Medicine, Juntendo University Faculty of Medicine, 2-1-1 Hongo, Bunkyo-ku, 113-8421 Tokyo, Japan

**Keywords:** Well leg compartment syndrome, Robot-assisted radical cystectomy, Lithotomy position, Fasciotomy

## Abstract

**Background:**

The indications for robot-assisted urologic surgeries have expanded due to their low invasiveness. However, complicated surgical procedures lead to prolonged surgical duration, requiring patients to remain in the lithotomy position for an extended time. Well leg compartment syndrome (WLCS) is a known severe postoperative complication related to the lithotomy position.

**Case presentation:**

We report a case of WLCS after robot-assisted radical cystectomy (RARC), in which the patient recovered without neurological sequelae. A 55-year-old, obese male who underwent RARC complained of right leg pain and paresthesia 3 h after the surgery that lasted for 481 min. Emergency evaluation revealed unilateral WLCS in the anterior and lateral compartments. Urgent fasciotomy was performed 4 h after symptom onset. He thereafter recovered completely and was discharged without any neuromuscular dysfunction.

**Conclusions:**

Early detection of WLCS, surgical treatment, and additional measures are crucial to prevent its life-threatening and/or disabling outcomes.

## Background

Compartment syndrome is a severe morbidity that can lead acutely to rhabdomyolysis and renal failure and chronically to neurogenic paralysis and permanent disability [1, 2]. It develops when excessive pressure builds up inside enclosed muscle compartments, mostly following traumatic events. Well leg compartment syndrome (WLCS) is defined as compartment syndrome caused by the intraoperative lithotomy position [1, 2].

Recently, robot-assisted urologic surgeries become more common than conventional surgeries due to their less invasiveness [3, 4]. However, since some procedures require a longer duration due to surgical complexity in the narrow pelvis, these surgeries often require patients to remain in the lithotomy position for a longer period, which may predispose to WLCS.

Here, we report a rare case of unilateral WLCS following robot-assisted radical cystectomy (RARC).

## Case presentation

A 55-year-old, 168-cm, 88.3-kg male with a body mass index (BMI) of 31.3 kg/m^2^ was scheduled to undergo RARC for bladder cancer. He had diabetes mellitus and a history of smoking.

After placement of a lower thoracic epidural catheter, general anesthesia was induced. Anesthesia was maintained with combined general and epidural anesthesia. The FloTrac^TM^ system (Edwards Lifesciences, Irvine, CA, USA) was used to achieve adequate fluid management. The patient was placed in the lithotomy position with stirrups (Levitator®, Mizuho Medical, Tokyo, Japan), after elastic stockings (ES; Comprinet Pro® Size 3, Terumo, Tokyo, Japan) and intermittent pneumatic compression (IPC) devices (Flowtron Excel®, Muranaka, Osaka, Japan) were applied to prevent deep vein thrombosis (DVT). Intraoperatively, phenylephrine was infused at 0.5–1 mg/h to maintain mean blood pressure (BP) above 60 mmHg. Heart rate was kept stable between 50 and 90 beats/min and SpO_2_ was maintained at 98% or more.

RARC was performed using the da Vinci Xi surgical system® (Intuitive Surgical Inc., Sunnyvale, CA, USA). Total cystectomy, extended pelvic lymph node dissection, and intracorporeal urinary diversion with an ileal conduit were performed uneventfully. However, the surgical duration was prolonged as long as 481 min due to expansion of lymph node dissection. Total blood loss, including urine volume, and total infusion volume were 300 ml and 5560 ml, respectively. The patient remained in the lithotomy position for 515 min, during which he remained in an extreme 25-degree Trendelenburg position for 201 min. He was extubated immediately after the surgery and was transferred to the intensive care unit.

**Fig. 1:**
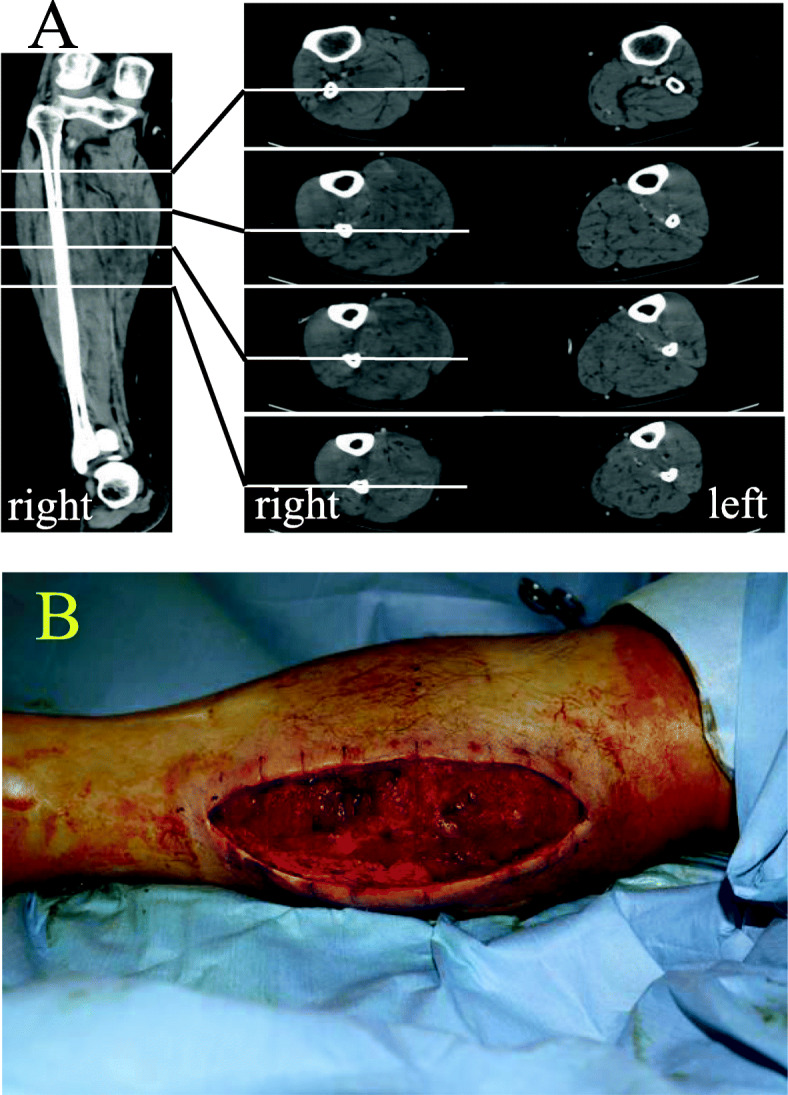
**a** Contrast-enhanced CT imaging of the lower limbs, and **b** the lateral aspect of the right lower limb after fasciotomies on both sides of the calf in the present case. Left and right panels of **a** present a coronal view of the right lower limb and axial views of bilateral lower limbs, respectively. Note that in **a**, the right lower limb shows an increase in size (i.e., swelling) and a decrease in contrast enhancement (i.e., hypoperfusion), compared with the left lower limb

At 3 h postoperatively, he complained of paresthesia and pain over the right calf. The epidural infusion was discontinued because the paresthesia might result from the effect of epidural analgesia. However, the paresthesia and pain were exacerbated 1 h after suspension of the epidural infusion. Urgent blood examinations revealed an elevated level of creatine kinase (CK) (15,800 U/l). Computed tomography (CT) revealed swelling of the right calf (Fig. 1a). He was diagnosed with WLCS and transferred back to the operating theater for urgent fasciotomy at 7 h postoperatively.

Compartment pressures of the right calf measured under general anesthesia with a needle manometer (TraWave®, Edwards Lifesciences, Irvine, CA, USA) were high at 38, 19, and 14 mmHg in the anterior, lateral, and posterior compartments, respectively. Fasciotomies performed on both sides of the calf reduced compartment pressures to 18, 14, and 12 mmHg, respectively (Fig. 1b). Subsequent hematological examinations indicated a return of CK to a normal level (218 U/l) by postoperative day 15.

After the fasciotomy, he required debridement procedures twice due to local infection, followed by a skin graft to compensate for the skin defect 43 days after RARC. He was finally discharged from hospital 78 days after RARC without any neuromuscular disorders and in an ambulatory condition.

## Discussion

**Fig. 2:**
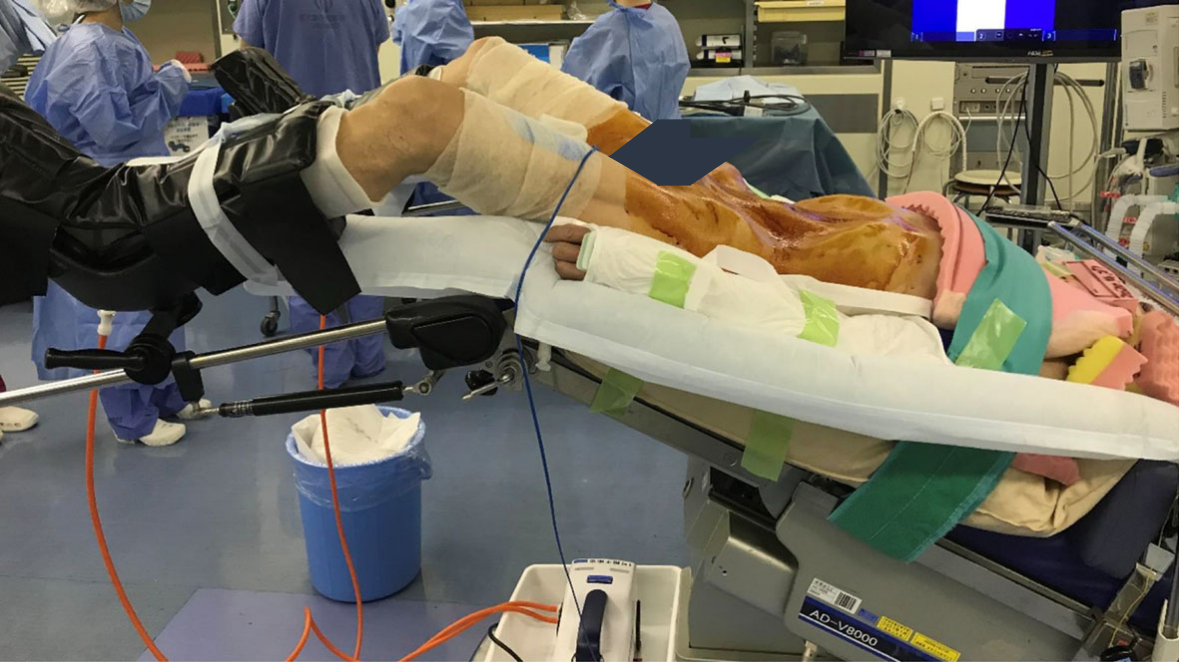
A typical lithotomy position with a 25-degree head-down tilt for robot-assisted radical cystectomy

WLCS related to the lithotomy position is a rare but potentially devastating complication [1, 2]. The lithotomy position, especially that with a head-down tilt, results in a decrease in static perfusion pressure to the lower limbs due to their positions elevated above the heart [1, 2, 5]. Because mean BP in the calf decreases by 0.78 mmHg for every 1-cm elevation above the heart [1], an estimated mean BP in the calf in our patient could have been 25–30 mmHg lower than systemic BP when he was placed in the 25-degree Trendelenburg lithotomy position (Fig. 2). Further, calf compartment pressures increase suddenly upon placement of legs in the lithotomy position, especially in that with a head-down tilt, due to direct mechanical compression from leg holders [6–8]. Thus, the combination of decreased perfusion pressures and increased compartment pressures results in a significant decrease in driving pressure to lower limbs, which is exaggerated by a head-down tilt [1, 2]. This can induce tissue ischemia, which causes hypoxic disruption of the capillary endothelium, interstitial edema formation secondary to fluid leaks, and thus, further increases in compartment pressures impeding arterial perfusion further. Compartment pressures can increase even further after adequate tissue perfusion is restored by repositioning of the legs as a result of reperfusion injury. These can eventually lead to WLCS [1, 2].

The incidences of WLCS in conventional abdominopelvic surgeries and robot-assisted radical prostatectomy (RARP) are reported to be 0.03% (1/3500) and 0.29% (9/3110) [5, 9], respectively. Compared with open procedures, laparoscopic and robotic procedures are supposed to increase a risk of WLCS as a result of prolonged surgical durations, greater degrees of head-down tilts, and reduced venous return in the lower limbs due to pneumoperitoneum [10]. In our case, RARC was performed, and consequently, the surgical duration was prolonged. Mean surgical durations of RARP and RARC at our institution are 170 min and 503 min, respectively (unpublished data). Prolonged surgical durations for RARC can predispose to WLCS.

Our patient had a high BMI. Obesity is a risk factor of WLCS [1, 2, 9]. In patients with a BMI above 25 kg/m^2^, ankle BP in the lithotomy position decreases linearly with an increasing BMI [11]. Meanwhile, calf compartment pressures in the lithotomy position increase linearly with an increasing BMI, reflecting bulky legs in obese patients [6], which may explain lower ankle BP in obese patients [11]. Although it remains unknown whether the uses of ES and/or IPC to prevent DVT predispose to WLCS [2], compression by ES generates a constant pressure over the calf [12], while reducing local blood flow [13]. Compression of the extremity by ES might have been too tight in our obese patient with bulky legs. Meanwhile, the use of IPC seemed appropriate because IPC attenuates the elevation of calf compartment pressures resulting from the lithotomy position [8]. We infused phenylephrine intraoperatively. In theory, the use of vasoconstrictor may help to prevent WLCS by preventing hypotension, or conversely, it may predispose to WLCS through peripheral vasoconstriction. However, no evidence has currently been identified to implicate the perioperative use of inotropes/vasoconstrictors in the etiology of WLCS [2].

A compartment pressure is normally around 0–10 mmHg. Reportedly, a compartment pressure exceeding 30 mmHg is an absolute indication for fasciotomy, and the golden time for this procedure is within 8 h of symptom onset [14, 15]. In our case, even though a compartment pressure exceeded 30 mmHg, the patient did not suffer from any neurological sequelae possibly owing to the urgent surgical treatment 4 h after symptom onset, well within the golden time.

**Fig. 3:**
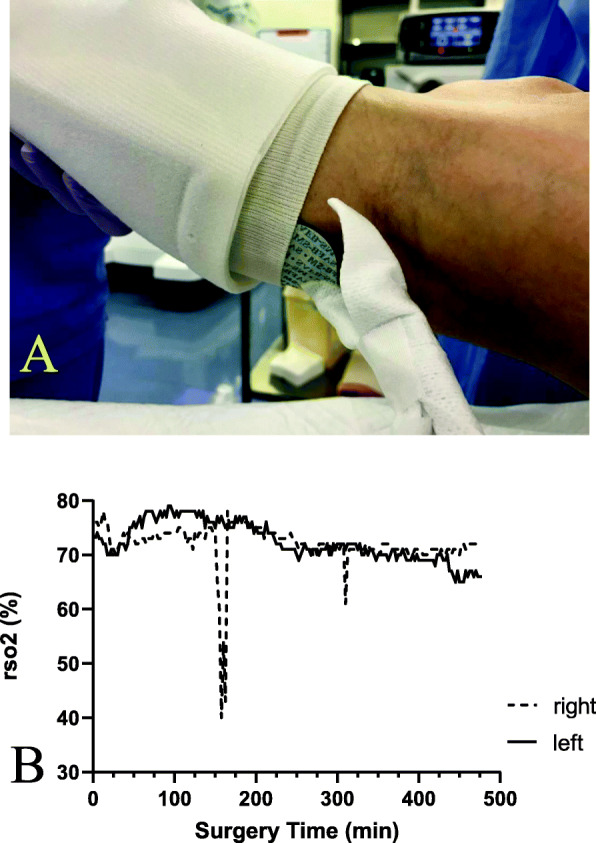
**a** A manner of regional oxygen saturation (rSO_2_) monitoring in bilateral lower limbs using a NIRS oximeter (INVOS 5100^TM^, Medtronic, Minneapolis, MN, USA), and **b** trends in bilateral rSO_2_ in a different patient. NIRS sensors are positioned on the skin surfaces of bilateral calves (**a**). Note that in **b**, rSO_2_ in the right lower limb (dotted line) showed a profound decrease, which began at around 150 min and lasted for about 15 min during pelvic lymph node dissection possibly due to excessive traction on iliac vessels

After experiencing this case, we have changed our practice. First, we now perform regular leg reperfusion/decompression procedures by returning the patient’s position from the head-down, lithotomy position to the horizontal, supine position every 3 h, based on a previous report showing that compartment pressures gradually increase with time during the lithotomy position, reaching above 30 mmHg within 3.5–6.0 h, but the pressures rapidly return to normal upon repositioning into the supine position [7]. Second, we now monitor tissue oxygen saturation of lower limbs using near-infrared spectroscopy (NIRS) with its sensors positioned on the skin surfaces of bilateral calves (Fig. 3a), as recently recommended [16, 17]. Actually, we detected hypoperfusion of a unilateral leg during pelvic lymph node dissection in a subsequent case (Fig. 3b), suggesting that a NIRS oximeter is useful to detect hypoperfusion of legs and to prevent WLCS.

Unknown is why WLCS developed unilaterally in our case. Clearly, some relevant risk factors alone, including a prolonged surgical duration, obesity, applications of ES/IPC, and the use of phenylephrine, cannot explain the laterality of WLCS. However, slight differences between both legs in their positioning and applications of ES/IPC to them over a prolonged period might have contributed to the development of unilateral WLCS. Alternatively, longer-lasting unilateral leg ischemia, compared to the case as shown in Fig. 3b, might have occurred during the expanded pelvic lymph node dissection. Practicing regular leg reperfusion/decompression procedures and applying tissue oxygenation monitoring might have prevented WLCS in our case.

## Conclusion

Early detection of perioperative WLCS from symptoms (e.g., leg pain) and signs (e.g., calf swelling) and early surgical treatment, if indicated, are crucial to prevent its life-threatening and/or disabling outcomes. Additional measures to prevent WLCS should be considered in patients undergoing prolonged surgeries in the lithotomy position.

## Data Availability

The datasets related to this report are available from the corresponding author on reasonable request.

## Abbreviations

WLCS: Well leg compartment syndrome
RARC: Robot-assisted radical cystectomy
BMI: Body mass index
ES: Elastic stockings
IPC: Intermittent pneumatic compression
DVT: Deep vein thrombosis
BP: Blood pressure
CT: Computed tomography
CK: Creatine kinase
RARP: Robot-assisted radical prostatectomy
NIRS: Near-infrared spectroscopy
rSO_2_: Regional oxygen saturation
